# Human Adipocytes Stimulate Invasion of Breast Cancer MCF-7 Cells by Secreting IGFBP-2

**DOI:** 10.1371/journal.pone.0119348

**Published:** 2015-03-06

**Authors:** Chen Wang, Chao Gao, Kui Meng, Haishi Qiao, Yong Wang

**Affiliations:** 1 State Key Laboratory of Chemistry for Life Science & Jiangsu Key Laboratory of Molecular Medicine, Medical School of Nanjing University, Nanjing 210093, China; 2 The Center for Clinical Reproductive Medicine, Jiangsu Province Hospital, Nanjing 210029, China; 3 The Gulou Hospital attached to Nanjing University, Nanjing 210008, China; INRS, CANADA

## Abstract

**Background and Aims:**

A better understanding of the effects of human adipocytes on breast cancer cells may lead to the development of new treatment strategies. We explored the effects of adipocytes on the migration and invasion of breast cancer cells both in vitro and in vivo.

**Methods:**

To study the reciprocal effects of adipocytes and cancer cells, we co-cultured human mature adipocytes and breast cancer cells in a system devoid of heterogeneous cell-cell contact. To analyze the factors that were secreted from adipocytes and that affected the invasive abilities of breast cancer cells, we detected different cytokines in various co-culture media. To study the communication of mature adipocytes and breast cancer cells in vivo, we chose 10 metastatic pathologic samples and 10 non-metastatic pathologic samples to do immunostaining.

**Results:**

The co-culture media of human MCF-7 breast cancer cells and human mature adipocytes increased motility of MCF-7 cells. In addition, MMP-2 was remarkably up-regulated, whereas E-cadherin was down-regulated in these MCF-7 cells. Based on our co-culture medium chip results, we chose four candidate cytokines and tested their influence on metastasis individually. We found that IGFBP-2 enhanced the invasion ability of MCF-7 cells in vitro more prominently than did the other factors. In vivo, metastatic human breast tumors had higher levels of MMP-2 than did non-metastatic tumor tissue, whereas adipocytes around metastatic breast tumors had higher levels of IGFBP-2 than did adipocytes surrounding non-metastatic breast tumors.

**Conclusions:**

IGFBP-2 secreted by mature adipocytes plays a key role in promoting the metastatic ability of MCF-7 breast cancer cells.

## Introduction

Adipocytes are the most abundant and perhaps the most active component of the breast cancer stroma [1,2]. When human breast cancer cells break through the basal membrane, they are exposed to adipocytes in the immediate tumor microenvironment as they traverse the underlying connective tissue towards the bloodstream. Recent accumulating evidence has reported that the tumor-adjacent adipose tissue is a key component of breast cancer progression. In addition, obesity has recently emerged as an independent negative prognostic factor for breast cancer independently of menopause status [3].

In addition to releasing free fatty acids (FFAs) for the regulation of energy balance, human mature adipocytes also secrete various bioactive proteins. The altered gene expression observed in breast cancer cells is determined by soluble factors produced in the tumor microenvironment, both by cancer cells and stromal adipocytes [4,5]. Several molecules that are produced by the adipocytes in the tumor microenvironment are metastasis-associated proteins, and different cytokines, such as MMP-11, PAI-1, collagen VI, IL-6, IL-1β, TNFa, leptin and IGF-1[6–11]. However, the mechanism of spontaneous cross talk between breast cancer cells and mature adipocytes is not yet understood. Some researchers have reported that adipocytes cultivated with cancer cells exhibit decreased adipocyte markers associated with the overexpression of matrix metalloproteinase-11 and pro-inflammatory cytokines such as interleukin (IL)-6, IL-1β and IL-8 [8,12]. The study of J.H. Kim found that palmitate increased the invasiveness of MDA-MB-231 cells in the adipocyte cultured medium, but protein components did not [13]. Therefore, determining which adipocyte-derived factors chiefly contribute to stimulating breast cancer cell progression still needs much more investigation.

Here, we have investigated whether the transient interaction between human breast cancer cells and human adipocytes can induce phenotypic changes in the cancer cell and affect its invasion ability. We provide evidence that such an interaction induces the secretion of endogenous cytokines by the adipocytes, which can enhance the malignant behavior of the breast cancer cells *in vitro* and *in vivo*. In addition, we found that the IGFBP-2 secreted by adipocytes is mainly responsible for the increased invasion ability of breast cancer cells.

## Materials and Methods

### Ethics Statement

The study was approved by the insititutional review board (CWO) of medical school of Nanjing university, China. All patients provided written informed consent.

### Cell culture

Adipose tissue was obtained from the abdominal subcutaneous fat of a female patient who was healthy and free from metabolic or endocrine diseases. The tissue was cut into 1-mm pieces and incubated with collagenase with shaking at 37°C for 1 hour. To enrich for adipocyte stem cells, large debris were removed from the supernatant by filtering it through 100-μm nylon mesh, followed by centrifugation over discontinuous Percoll (Sigma-Aldrich) density gradients. We called the adherent adipocyte stem cells pre-adipocytes and cultured them in high glucose Dulbecco's modification of Eagle's medium (DMEM) supplemented with 10% fetal bovine serum (FBS). The cell phenotype was confirmed as CD44-, CD29-, and CD105-positive by flow cytometry.

5×10^5^ human pre-adipocytes were seeded into the bottom well of the co-cultures system which had the area of 962mm2. The pre-adipocyte differentiation medium was purchased from ScienCell (Product Code PADM, Catalog Number 7221). The induction medium was changed every two days, and cells were fixed and stained with oil red O solution after 5 days. On the 10th day, more than 80% of human pre-adipocytes were filled with multiple lipid droplets. The human mammary carcinoma cell lines MCF-7 and MDA-MB-231 were obtained from the Cell Bank of the Chinese Academy of Sciences. Cells were maintained in high glucose DMEM (Invitrogen-GIBCO) containing 110 mg/L sodium pyruvate, 2 mg/L pyridoxine hydrochloride, and 2 g/L sodium bicarbonate. The medium was supplemented with 10% FBS, penicillin (100 U/ml), and streptomycin (100 mg/ml). Cell cultures were maintained at 37°C in 5% CO2. For the authentication of MCF-7 and MDA-MB-231 cell lines, immunoblotting was used to confirm that estrogen receptor (ER) a and E-cadherin were positive in MCF-7 cells, and vimentin was positive in MDA-MB-231 cells.

### Oil red O stain

The intracellular lipid content was evaluated with the lipophilic dye oil red O [14]. Media was removed and cells were fixed with 4% paraformaldehyde for 15 minutes. The oil red O stock solution (300 mg/L in isopropanol) was diluted 3:2 with distilled water and filtered immediately before use. Samples were then stained for 1 hour with the working solution of oil red O and then washed twice with distilled water for 15 min. An Olympus CKX41 phase contrast microscope was used to examine random areas of the oil red O-stained cells. Cells were dehydrated and isopropanol was added to each well. OD values were detected by a MD-SpectraMax M5 at 510 nm.

### Co-culture and category of conditioned media

To obtain conditioned medium (CM) from co-cultures, cancer cells and adipocytes were co-cultured using a 6-well transwell culture system (1-μm pore size; Millipore PIRP30R48). A total of 1.5X10^5^ MDA-MB-231 cells or 3X10^5^ MCF-7 cells were seeded in the top chamber of the transwell system in DMEM with mature adipocytes in the bottom chamber. The conditioned medium of lower chamber of the transwell was collected after 2 days and was called cancer-associated adipocytes conditioned medium (CAA-CM). CM obtained from adipocytes that had been cultivated for 2 days was called adipocytes conditioned medium (A-CM). Routine DMEM with 10% FBS was called C-CM. To evaluate the effect of different conditioned medium on tumor cell invasion, we used CAA-CM, A-CM, and C-CM to stimulate MCF-7 cells for 48 hours.

CM obtained from the co-culture of IGFBP-2 knock-out adipocytes and MCF-7 cells was called CAA-CM A-. CM obtained from the co-culture of IGFBP-2 knock-out MCF-7 cells and adipocytes was called CAA-CM M-.

### Cell migration and invasion

Scratch assay: MCF-7 breast cancer cells were pre-treated individually with CAA-CM, A-CM and C-CM for 24 hours in 12-well plates. Then, cell proliferation was blocked by a 30 minute pretreatment with mitomycin C (40 μg/ml). A scratch was then made in each well using a 200-μl pipette tip, and cells were maintained in DMEM with 1% serum. The distances between the two edges were scaled for five positions after 24 hours. The experiment was replicated for three times and triplicate wells were used every time.

Invasion assays: After incubation in CAA-CM, A-CM or C-CM for 24 hours, breast cancer cells were trypsinized and resuspended in medium without FBS. When individual adipokines were used in invasion assays there was no FBS in the medium. The upper chambers of the transwells (8.0-μm membrane pores; Costar) were coated with 2.5 mg/ml Matrigel (BD Biosciences LOT: 356234) and incubated at 37°C for 30 minutes. A total of 10^5^ cells in 200 μl of medium without FBS were added to the upper chamber and allowed to migrate toward the bottom chamber, which contained medium with 20% FBS as a chemo-attractant. After 24 hours, the cells on the top surface of the membranes were removed using a cotton swab, and cells on the underside were fixed in 3.7% paraformaldehyde (PFA) and stained with crystal violet. Quantification was performed by dissolving crystal violet with 10% acetic acid, and samples were detected with a microplate reader at 595 nm. Pictures were taken with phase contrast microscope at a 200-fold magnification. Experiments were performed in duplicate and repeated at least three times with consistent results. Invasion assays were also conducted with MCF-7 cells that had been previously cultivated for 1 day with IGFBP-2 (680 pg/ml), PDGF-BB (210 pg/ml), IL-6sR (110 pg/ml) and TNF-a (529 pg/ml) individually.

### RNA isolation and RT-qPCR

Total RNA was isolated using Trizol (Invitrogen) according to the manufacturer’s instructions. RNA yields were measured using a NanoDrop ND-2000 instrument. First-strand cDNA was synthesized from total RNA (1 μg) by reverse transcription using oligo-dT primers and Superscript II reverse transcriptase (Invitrogen), according to the manufacturer’s instructions. Quantitative real-time PCR was performed in a 20 μl mixture containing 1 μl of the cDNA preparation, 10 μl 2X SYBR Green Premix Ex Taq (Takara, DRR041S), and 1 μM primer on an ABI Step One PCR Instrument. The thermal profile for the real-time PCR was: 5 minutes at 95°C followed by 40 cycles of 30 seconds at 95°C and 1 minute at 60°C. Each sample was tested in triplicate. Threshold values were determined for each sample/primer pair, and the average and standard error were calculated. The PCR products were verified by melting curve analysis as well as by 1.8% agarose gel electrophoresis of the PCR product. GAPDH was used as an internal standard of mRNA expression.

### Measurement of adipocyte-derived cytokines

A RayBio Human Adipokine Antibody Array I was used to analyze culture medium from the co-culture according to the protocol supplied by the manufacturer. The glass chip was blocked for 30 minutes with protein-free blocking buffer (RayBiotech, Inc.), and 1 ml of sample was added per sub array and incubated overnight at 4°C. The biotin-conjugated antibodies and fluorescent dye-conjugated streptavidin (Cy3 equivalent) supplied in the kit were both diluted in the protein-free blocking buffer and incubated successively with the glass chip for 2 hours at room temperature before detection using a LuxScan10K-A microarray scanner (Capital Bio). Spot intensities were quantified using LuxScan 3.0 software and normalization to the background. All procedures were performed by CapitalBio Corporation, Beijing, P.R. China (http://www.capitalbio.com). According to the manufacturer's introductions, the threshold for a significant difference in expression was defined as: any ≥ 1.5-fold increase or ≤ 0.65-fold decrease in signals detected from arrays for a single cytokine between samples. TNF-a adipokine levels in culture supernatants were analyzed using an ELISA kit (R & D), and other adipokine concentrations were converted by the concentration of TNF-a according to the signal value change given by the chip results. Recombinant human IGFBP-2, PDGF-BB, IL-6sR and TNF-a were purchased from Peprotech and dissolved in auxiliary solvent.

### SiRNA transfection

We designed three siRNA sequences targeting different sites to knock down human IGFBP-2 mRNA. They were transfected into MCF-7 cells using Lipofectamine 2000 (Invitrogen) and the expression levels of IGFBP-2 mRNA were assessed by quantitative RT-PCR. The sequence with the best interfering effects which had 60% mRNA knockdown was tested in invasion assays. The sense sequence was 5’-GGAGCAGGUUGCAGACAAUTT-3’ and the antisense sequence was 5’-AUUGUCUGCAACCUGCUCCTT-3’. MCF-7 cells and adipocytes were transfected with either the siRNA sequence or the negative control mimic using Lipofectamine 2000. Prior to transfection, cells that had reached 80–85% confluence in 6-well culture dishes were added to 1500μl growth medium without antibiotics. Prior to the complex preparation, 100 picomolar siRNA was diluted in 250 μl of medium without serum, and 5 μl of Lipofectamine 2000 was diluted in 250 μl of medium without serum. These solutions were mixed gently and incubated for 20 min at room temperature. Then, 500 μl of the resulting complexes was added to each well containing cells and medium without serum or antibodies. We incubated the cells at 37°C in a 5% CO_2_ incubator for 48 h and then tested the efficiency of mRNA expression by reverse transcription and quantitative PCR (RT-qPCR).

### Western blotting and immunostaining

Western blotting was performed following established protocols [15]. The following antibodies were used: IGFBP-2 (1:1000, Abcam4243), MMP-2 (1:1000, Abcam86607), and GAPDH (1:1000, BD Biosciences).

Immunostaining was performed on 5-μm sections of formalin-fixed, paraffin-embedded tumor tissues. The breast tumor tissues were provided by the pathology department of Nanjing Gulou Hospital. Tissue sections were deparaffinized in xylene and hydrated in a graded sequence of ethanol solutions. For antigen retrieval, sections were pretreated in a microwave in citrate buffer (pH 6.0). After cooling, nonspecific binding was blocked with diluted serum (4% normal goat serum) followed by incubation with antibodies against IGFBP-2 (1:300, Abcam109284) and MMP-2 (1:1000, Abcam 86607) at room temperature in a humidified chamber for 2 hours. Negative controls were analyzed on adjacent sections incubated without primary antibody. After incubation with the primary antibody, sections were washed with PBS and subsequently treated using the corresponding biotinylated secondary antibody from an Ultravision ONE kit (Thermo Fisher Scientific) according to the manufacturer’s protocol. Peroxidase activity was visualized using 3, 3’-diaminobenzidine, and sections were counterstained with hematoxylin.

### Statistical analysis

Quantitative RT-PCR and cell migration assays were performed in triplicate, and each experiment was repeated three to five times. The data shown are presented as the mean ± S.D. of at least three independent experiments. Statistical significance was considered at P <0.05 using Student’s t test.

## Results

### Medium conditioned by co-cultures of human mature adipocytes and cancer cells induces the cell migration and invasion in breast cancer cells

**Fig 1:**
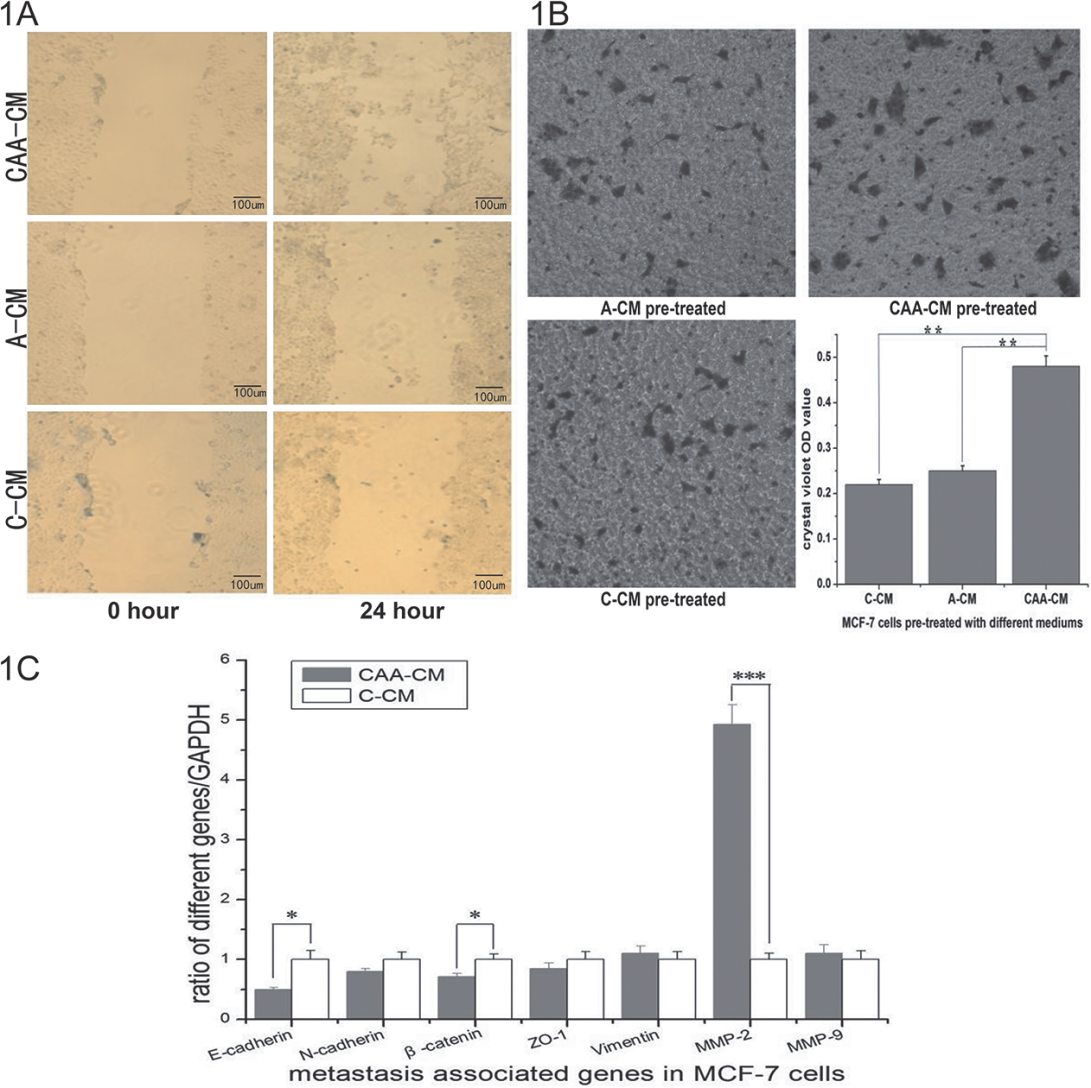
Function of the medium generated from the co-culture of MCF-7 cells and mature adipocytes. 1A. MCF-7 cells were stimulated with different conditioned media for 24 hours, and then mitosis was inhibited by incubation with mitomycin C for 30 minutes. C-CM and A-CM pre-treated MCF-7 cells showed no change in the scratch width, whereas CAA-CM pre-treated cells had 26%±1.5% wound healing and many cells were found scattered in the middle of the scratch (100 × magnification). 1B. MCF-7 cells treated with CAA-CM showed greater invasion ability than A-CM and C-CM. Quantification was performed by dissolving crystal violet with 10% acetic acid and detecting the OD values with a microplate reader at 595 nm. Pictures were taken at 200 × magnification. The data shown are the means of triplicate wells, and the error bars represent the S.D (**: P<0.01). 1C. Quantification of E-cadherin, N-cadherin, β-catenin, ZO-1, Vimentin, MMP-2 and MMP-9 in MCF-7 cells treated with different media. These mRNAs were detected by quantitative RT-PCR and normalized to GAPDH. The primers used for quantitative PCR are listed in Table 1. The ratios of signal were analyzed for statistical significance using the t-test (*: P<0.05, ***: P<0.001).

To address the effect of adipocyte-secreted factors on the breast cancer cells, we used conditioned media from 2-day-old homotypic cultures or co-cultures to stimulate MCF-7 cells that had not been previously exposed to any conditioned media. We used MCF-7, which is a cancer cell line with low metastatic potential, for scratch assays and found that medium conditioned by co-cultures (CAA-CM) significantly accelerated the closure of the cell-cleared area compared to unconditioned control medium (C-CM) (Fig. 1A). In invasion assays, CAA-CM caused a significant increase in the number of invasive MCF-7 cells, whereas cells treated with A-CM or control medium showed little invasion (Fig. 1B). This observation suggested that co-cultures might exhibit emergent properties that were not present in the homotypic cultures. To determine the mechanism of advanced migration and invasion ability in breast cancer cells, we chose to assess the mRNA expression levels of several metastasis-associated genes. MMP-2 was remarkably up-regulated whereas E-cadherin was down-regulated in MCF-7 cells treated with CAA-CM compared to those treated with C-CM (Fig. 1C). Taken together, these data suggest that the co-culture of breast cancer cells with mature adipocytes might cause the secretion of some soluble factor that increases breast cancer cell migration and invasion.

**Table 1:** Primers used for Q-PCR amplification of human breast cancer cells.

Gene	Primer
E-cadherin	R: 5- TGCCCCATTCGTTCAAGTAG-3
	F: 5- AATCTGAAAGCGGCTGATACTG-3
N-cadherin	R: 5- CAGCGTTCCTGTTCCACTCATA-3
	F: 5- TGTTTTGGACCGAGAATCACC-3
β-catenin	R: 5- ATCCACCAGAGTGAAAAGAACG-3
	F: 5- GCCACAAGATTACAAGAAACGG-3
ZO-1	R: 5- ATGGTCGGGCAGAACTTGTAT-3
	F: 5- TTTGGCGAGAAACGCTATGA-3
Vimentin	R: 5- CGTGATGCTGAGAAGTTTCGTT-3
	F: 5- TCTGGATTCACTCCCTCTGGTT-3
MMP-2	R: 5- GCTTCCAAACTTCACGCTCTTC-3
	F: 5- AATGCCATCCCCGATAACCT-3
MMP-9	R: 5- TCAGTGAAGCGGTACATAGGGT-3
	F: 5- CGAACTTTGACAGCGACAAGA-3
GAPDH	R: 5- GGAAGATGGTGATGGGATT-3
	F: 5- AACGGATTTGGTCGTATTG-3

### Co-cultivated adipocytes exhibit extensive phenotypic changes

**Fig 2:**
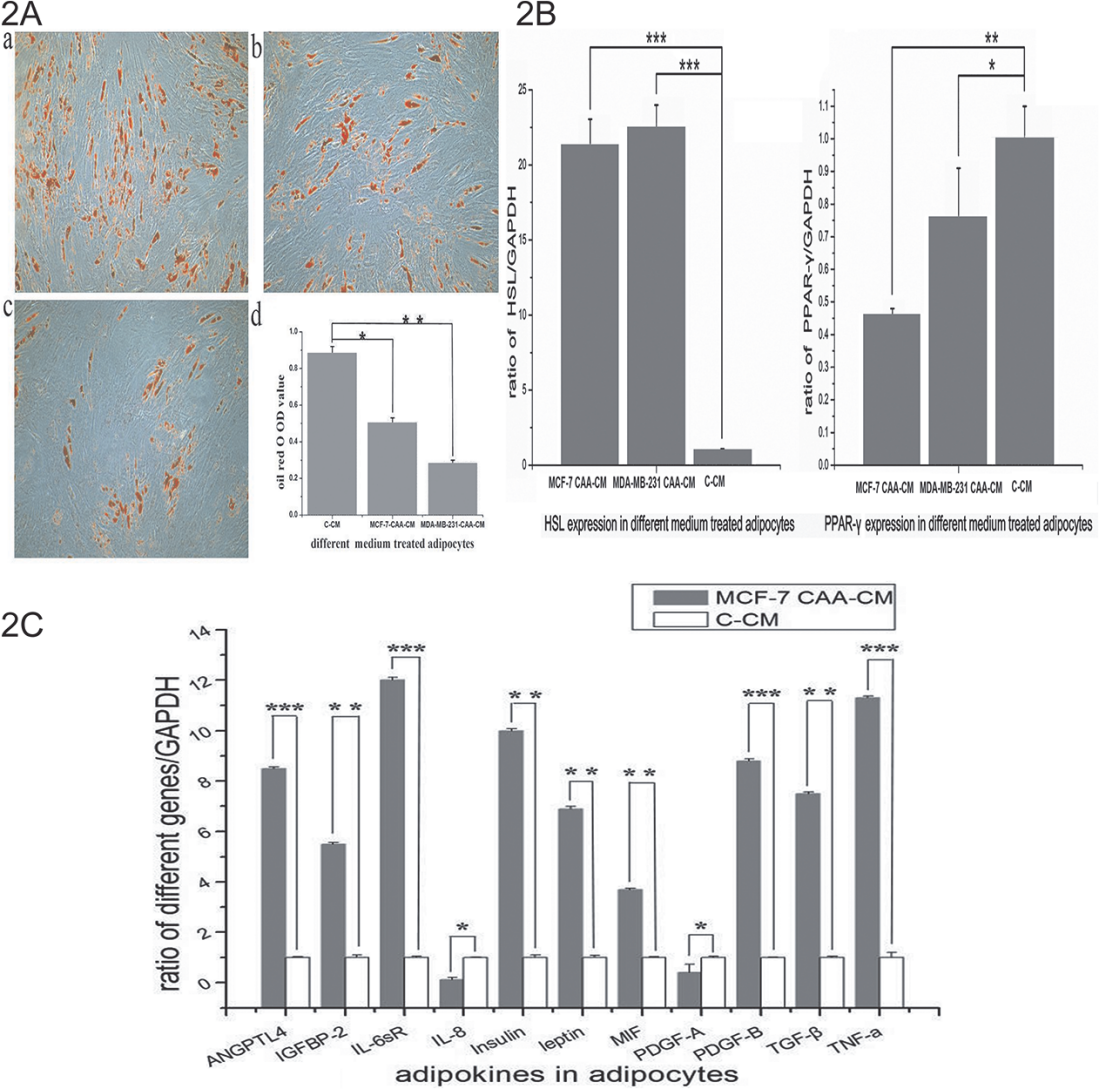
Co-culture changes the phenotype of human mature adipocytes. 2A. a—Human mature adipocytes treated with control CM. b—Human mature adipocytes treated with MDA-MB-231 CAA-CM. c—Human mature adipocytes treated with MCF-7 CAA-CM, 200 × magnification. d—Quantification was performed by detecting oil red OD values with a microplate reader at 500 nm. 2B. In co-cultivated adipocytes, peroxisome proliferator-activated receptor gamma (PPAR-γ) was decreased, whereas hormone-sensitive lipase (HSL) was increased at the mRNA level. 2C. Reverse transcription quantitative PCR was used to demonstrate genes in adipocytes that were up-regulated in MCF-7 CAA-CM in the chip assay. The primers used for quantitative PCR are listed in Table 3 (*: P<0.05, **: P<0.01, ***: P<0.001).

After confirming their contribution to tumor cell invasion, we aimed to determine what happened to adipocytes after co-culture. Our results showed that cross-talk between the two cell populations is necessary to observe the phenotype of co-cultivated adipocytes. As shown in Fig. 2A, mature adipocytes treated with co-cultivated medium exhibited a significant decrease in lipid accumulation, and this phenomenon was especially dramatic in the case of MDA-MB-231 CAA-CM- (Fig. 2A) compared to C-CM-treated adipocytes. MDA-MB-231 is an estrogen-negative, highly metastatic human breast cancer line, whereas MCF-7 is an estrogen-positive, human breast cancer line with low metastatic potential. The CAA-CM treated adipocytes lost the classical terminal differentiation marker peroxisome proliferator-activated receptor-γ (PPAR-γ) to some extent and showed a significant increase in hormone-sensitive lipase (HSL) (Fig. 2B).

Next, we evaluated the impact of co-culture on the expression of 62 adipokines in cell culture supernatant through protein microarray analysis, and we then further analyzed the adipokines that exhibited altered expression levels. In the chip assay, 12 adipokines were increased and 6 adipokines were decreased in MCF-7 CAA-CM compared to A-CM (Table 2). The increased proteins were angiopoietin like 4 (ANGPTL4), insulin-like growth factor binding protein-2 (IGFBP-2), interleukin-6 soluble receptor (IL-6sR), interleukin-8 (IL-8), insulin, leptin, macrophage migration inhibitor factor (MIF), platelet derived growth factor-AA (PDGF-AA), PDGF-AB, PDGF-BB, transforming growth factor-β (TGF-β), tumor necrosis factor-a (TNF-a).Among them, the up-regulation of IGFBP-2 and PDGF-BB was greater than ten-fold. The decreased proteins were adiponectin, angiopoietin-1, human neutrophil-activating peptide 78 (ENA-78), interferon-γ-inducible protein-10(IP-10), monocyte chemotactic protein 3 (MCP-3), regulated upon activation normal T-cell expressed and secreted factor (RANTES). Five proteins were elevated in MDA-MB-231 CAA-CM (data not shown), and they were different with proteins elevated in MCF-7 CAA-CM except PDGF-AA. At the mRNA level in adipocytes, a dramatic up-regulation of IL-6sR and TNF-a was noted in the adipocytes co-cultivated with MCF-7 cells (Fig. 2C). We chose IGFBP-2, PDGF-BB, IL-6sR and TNF-a as candidate factors because their protein concentration in MCF-7 CAA-CM and their gene expression in adipocytes were both elevated.

**Table 2:** Human-specific adipokine chip found several changes at protein level in co-culture medium.

	MCF-7 CAA-CM	A-CM	fold change
up-regulated proteins			
ANGPTL4	6.90	1.47	4.70
IGFBP-2	45.61	2.39	19.06
IL-6 sR	3.36	1.01	3.32
IL-8	4.27	2.16	1.98
Insulin	5.89	3.80	1.55
Leptin	17.35	2.01	8.64
MIF	7.33	1.08	6.79
PDGF-AA	44.15	6.63	6.66
PDGF-AB	12.60	0.95	13.22
PDGF-BB	32.73	1.28	25.52
TGF-β	5.31	2.66	1.99
TNF-a	4.57	2.73	1.67
down-regulated proteins			
Adiponectin	0.91	1.51	0.60
Angiopoietin-1	5.93	21.46	0.28
ENA-78	32.94	52.55	0.63
IP-10	2.04	4.12	0.49
MCP-3	35.33	131.32	0.27
RANTES	21.22	44.45	0.47

Every signal value in MCF-7 CAA-CM or A-CM was divided by that in C-CM which was DMEM containing 10% FBS but never having contacted with any cells. The number showed in the middle two columns was ratios that took signal value of C-CM as 1.

**Table 3:** Primers used for Q-PCR amplification of human mature adipocytes.

Gene	Primer
HSL	R: 5- CCTGTCTCGTTGCGTTTGT-3
	F: 5- ACATAGGGATGCTTCTATGGC-3
PPAR-γ	R: 5- AGGACTCAGGGTGGTTCA-3
	F: 5- AGGAGCAGAGCAAAGAGG-3
ANGPTL4	R: 5- CCCTTGGTCCACGCCTCTA-3
	F: 5- ACGGTGACTCTTGGCTCTGC-3
IGFBP-2	R: 5- GTCTACTGCATCCGCTGGGT-3
	F: 5- GCAAGGGTGGCAAGCATC-3
IL-6sR	R: 5- TGACCGTTCAGCCCGATA-3
	F: 5- AGCCGTGCCAGTATTCCC-3
IL-8	R: 5- GTGAGGTAAGATGGTGGC-3
	F: 5- TGTGGGTCTGTTGTAGGG-3
Insulin	R: 5- ACAATGCCACGCTTCTGC-3
	F: 5- CAGCCTTTGTGAACCAACACC-3
Leptin	R: 5- GAGGAGACTGACTGCGTGT-3
	F: 5- CTGTGCGGATTCTTGTGG-3
MIF	R: 5- GGAGTTGTTCCAGCCCACATT-3
	F: 5- ACCAGCTCATGGCCTTCGG-3
PDGF-A	R: 5- TTGGTTGACGCATAGTTC-3
	F: 5- ACACCTCCTCGCTGTAGT-3
PDGF-B	R: 5- ATTAAATAACCCTGCCCACA-3
	F: 5- CCTCATAGACCGCACCAAC-3
TGF-β	R: 5- GTGATGGACGGGAAAGACA-3
	F: 5- TTGAGCCCTCTAACTGAACG-3
TNF-a	R: 5- TGAAGAGGACCTGGGAGTAGAT-3
	F: 5- CGAGTGACAAGCCTGTAGCC-3
GAPDH	R: 5- GGAAGATGGTGATGGGATT-3
	F: 5- AACGGATTTGGTCGTATTG-3

### MCF-7 cell metastatic potential induced by Coculture Conditioned Medium depends on active IGFBP-2 synthesized by adipocytes

**Fig 3:**
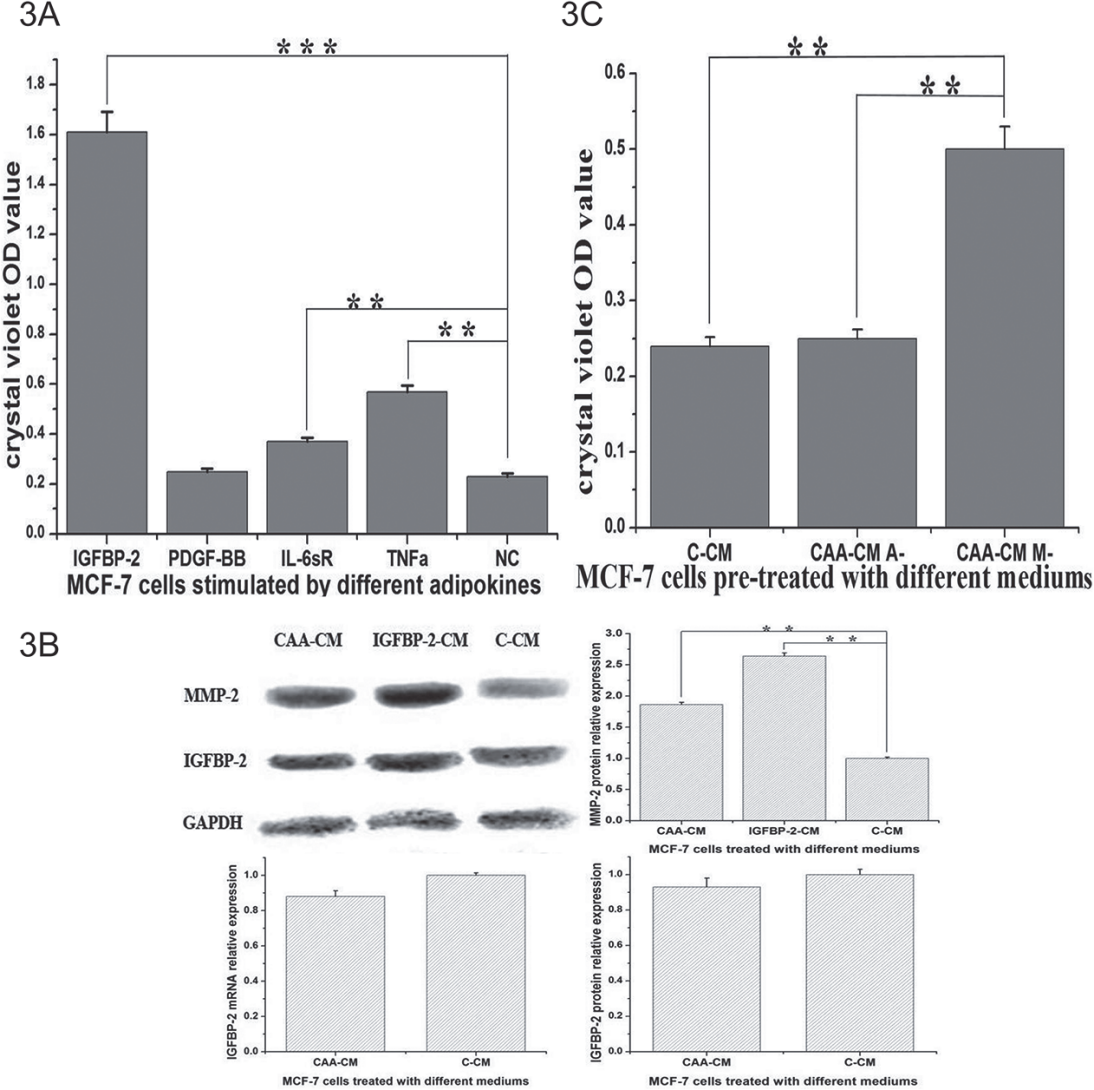
The role of IGFBP-2 in the communication of MCF-7 cells and adipocytes. 3A. MCF-7 cells were digested to put into transwells after incubation with IGFBP-2 (680 pg/ml), PDGF-BB (210 pg/ml), IL-6sR (110 pg/ml) and TNF-a (529 pg/ml) separately for 24 hours. IGFBP-2 enhanced the invasion ability of breast cancer cells most prominently at 680 pg/ml. 3B. We added IGFBP-2 to normal DMEM at 680 pg/ml and called it IGFBP-2-CM. CAA-CM and IGFBP-2-CM induced higher MMP-2 expression in MCF-7 cells than did C-CM. IGFBP-2 was not elevated in MCF-7 cells treated with CAA-CM at the mRNA or protein level. 3C. Medium obtained from the co-culture with IGFBP-2 knock-out adipocytes did not induce the invasion of the cancer cells, whereas the medium obtained from the co-culture of IGFBP-2 knock-out breast cancer cells were able to induce invasion (*: P<0.05, **: P<0.01, ***: P<0.001).

Cell metastatic potential may be induced by several cytokines secreted by adipocytes, so we tested the effect of four candidate factors at the concentration found by chip assay. IGFBP-2 (680 pg/ml) enhanced the metastasis of MCF-7 cells more prominently than other adipokines such as PDGF-BB (210 pg/ml), IL-6sR (110 pg/ml) and TNF-a (529 pg/ml) in the invasion assays (Fig. 3A). Furthermore, IGFBP-2 was the second most increased adipokine, with a 19.06-fold change in CAA-CM compared to A-CM as assessed by the adipokine chip assay. The protein PDGF-BB, which had the largest change in expression between CAA-CM and A-CM, did not demonstrate a significant role in promoting invasion at 210 pg/ml. To further address whether IGFBP-2 in CAA-CM might be secreted by adipocytes, we detected IGFBP-2 in MCF-7 cells at the mRNA and protein level (Fig. 3B). The fact that IGFBP-2 was not elevated in MCF-7 cells treated with CAA-CM at either the mRNA or protein level indicates that the concentration of IGFBP-2 in CAA-CM was secreted by adipocytes and not MCF-7 cells.

We used CAA-CM and CM plus IGFBP-2 (680 pg/ml) to stimulate MCF-7 cells, and found that both treatments induced higher MMP-2 expression at the protein level in MCF-7 cells than did C-CM (Fig. 3B). This result confirmed the hypothesis that CAA-CM-derived IGFBP-2 can increase MMP-2 signaling in breast cancer cells. We designed siRNA sequences to knock out IGFBP-2 mRNA, and siRNA sequences targeting different sites in human IGFBP-2 cDNA were transfected into MCF-7 cells. The expression level of IGFBP-2 mRNA was assessed by quantitative RT-PCR, and the sequence with the best interfering effect was used for the following test. While medium obtained from the co-cultures of IGFBP-2 knock-out breast cancer cells stimulated the invasion of breast cancer cells, co-culture medium from IGFBP-2 knock-out adipocytes did not induce cancer cell invasion (Fig. 3C). Taken together, these data suggest that the co-culture of breast cancer cells with adipocytes induces the production of IGFBP-2 by adipocytes, which then stimulates the invasion of breast cancer cells.

### IGFBP-2 increases breast tumor metastasis *in vivo*

**Fig 4:**
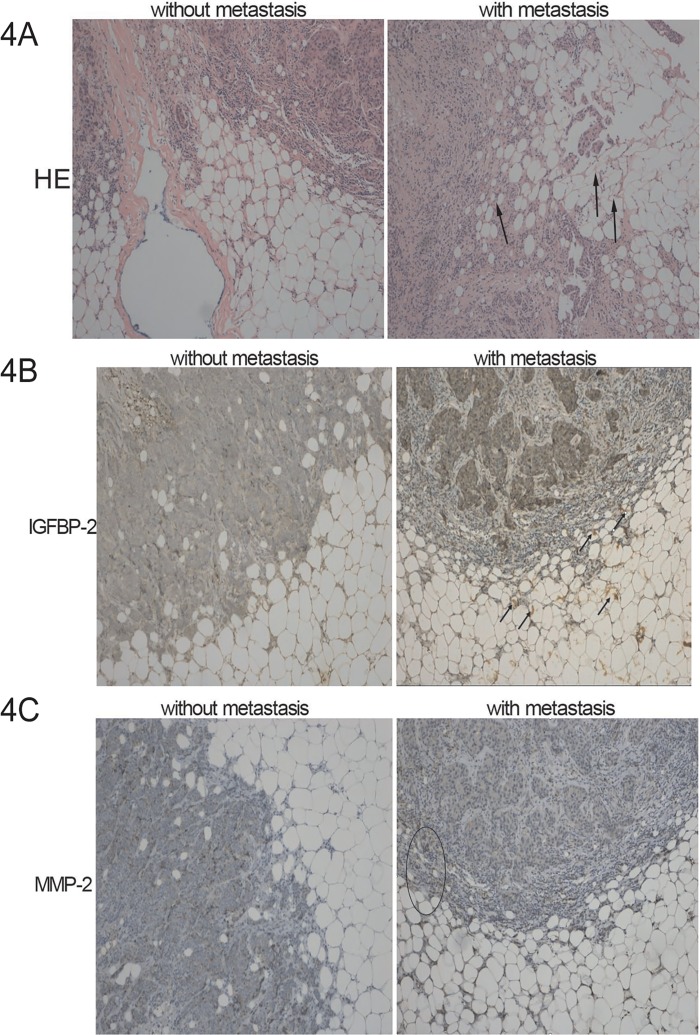
Immunohistochemical staining of IGFBP-2 in human adipocytes and MMP-2 in human breast tumors. 4A. Sections of metastatic and non-metastatic breast tumors and surrounding adipocytes were stained by hematoxylin and eosin (magnification 100 ×). Peritumoral adipocytes from infiltrant metastatic mammary tumors appear smaller and more spindle-shaped. 4B. Representative microphotographs show IGFBP-2 in human adipocytes around metastatic and non-metastatic breast tumors. Human adipocytes around metastatic breast tumors had higher levels of IGFBP-2 than did those around non-metastatic primary tumors. Arrowheads indicate IGFBP-2 positive cells (magnification 100 ×). 4C. Representative microphotographs show MMP-2 in metastatic and non-metastatic human breast tumors. Metastatic human breast tumors had higher levels of MMP-2 than did non-metastatic tumor tissue. Ellipse indicates MMP-2 positive cells at the edge of tumor (magnification 100 ×). The negative control was obtained by omitting the primary antibody.

We chose 10 metastatic pathologic samples and 10 non-metastatic pathologic samples to be stained by hematoxylin and eosin. We found that the adipocytes around metastatic tumors showed dramatic morphological changes compared with those surrounding non-metastatic tumors. They looked smaller and more spindle-shaped, especially when the tumor cells had broken through the basement membrane. We next aimed to determine the cellular origin and distribution of IGFBP-2 in metastatic and non-metastatic mammary tumor samples. We analyzed by immunohistochemistry the presence of MMP-2 in tumor cells and IGFBP-2 in the adipocytes around tumor cells in samples of human ductal infiltrant mammary tumors. As Fig. 4B shows, human adipocytes around metastatic breast tumors had higher levels of IGFBP-2 than did those around non-metastatic primary breast tumors.

Metastatic human breast tumors had higher levels of MMP-2 than did non-metastatic tumor tissue. In addition, MMP-2 was increased when tumor cells had invaded the adipocyte-containing stroma surrounding a primary tumor, as would be predicted from our observations. Thus, the communication between adipocytes and tumor cells may be responsible for the activation of MMP-2 in tumor cells and may durably permit the successful colonization of a wider area at the tumor edge.

## Discussion

The interactions between breast cancer cells and the adipocytes of the tumor microenvironment are complex and reciprocal [16,17]. Studying the crosstalk of cancer cells and mature adipocytes in breast cancer is of major clinical importance. Here, we have shown that transient interactions between human breast cancer cells and human mature adipocytes in vitro can increase cancer cell malignancy and expand the metastatic range of cancer cells. Specifically, the interaction of human breast carcinoma cells with mature human adipocytes induces the aberrant secretion of active IGFBP-2 by the adipocytes. This transient exposure of cancer cells to adipocyte-derived IGFBP-2 then durably increases metastasis even when the two cell types are no longer interacting. Thus, tumor cells traversing through connective tissue may exploit their transient interaction with mature adipocytes in order to promote the metastatic process.

In the present study, we induced human pre-adipocytes into human mature adipocytes and co-cultured them with human MCF-7 breast cancer cells without direct contact. The co-culture supernatants of human mature adipocytes co-cultured with MCF-7 cells led to the increased invasiveness of MCF-7 cells, which is an ER-positive breast cancer cell line with low metastatic potential. Similar results were obtained with the MDA-MB-231 cells, which are highly metastatic and ER-negative (data not included). When cancer cells were grown in the presence of CM obtained from adipocytes that had never been co-cultivated with cancer cells, their invasive capacity did not increase. These results revealed that the interaction between cancer cells and mature adipocytes can generate emergent system properties that are not observed when the cells are cultured separately. Based on some previous studies [4,8,18], we studied some metastasis-associated genes expression in MCF-7 cells and found that the up-regulation of MMP-2 was significantly correlated with the in vitro invasiveness of MCF-7, as shown in Fig. 1C. This finding may be explained by the fact that MMP-2, which is a type of matrix metalloproteinase, promotes the invasive ability of cancer cells through the dissolution of the extracellular matrix and by releasing an active fragment that stimulates cellular migration [19,20]. At the same time, the down-regulation of E-cadherin demonstrates the process of EMT (epithelial-mesenchymal transition), which is a complex multistage event accompanied by the modulation of genes associated with cytoskeletal and adhesion molecules [21]. We therefore proposed that the short term co-culturing of cancer cells with adipocytes without direct contact may change some factors in the medium and lead to appearance of a mesenchymal phenotype in cancer cells. We then decided to focus on how the adipocytes can increase the metastatic ability of breast cancer cells without the need for direct cell-cell contact.

It has been previously proposed that cancer cells can induce cancer associated characteristics in mature adipocytes which promote tumor progress; these adipocytes were called cancer-associated adipocytes (CAA) [8]. Andarawewa KL et al. found that invading breast cancer cells altered the adjacent adipocytes and decreased their differentiation into mature adipocytes, which was associated with decreased PPARγ expression [22]. J. Guerrero et al. found that soluble factors generated by the MDA-MB-231 and MCF-7 cancer cell lines inhibit the expression of PPARγ in human mammary adipocytes [23]. As expected from these studies, our experiment showed that delipidation was observed in adipocytes co-cultured in the presence of breast cancer cells compared with those cultured alone. Our RT-QPCR result showed that PPARγ, which is associated with mature adipocytes, was decreased in adipocytes co-cultivated with MDA-MB-231 and MCF-7 cells. The result was compatible with the speculation that this cross-talk between cancer cells and mature adipocytes dedifferentiated adipocytes. Suman K. Das et al. reported that HSL activity was significantly higher in the visceral white adipocyte tissue (WAT) of cancer patients compared with individuals who did not have cancer [24]. Mikael Rydén et al. proved that HSL stimulated the lipolysis of adipocytes differentiated from human mesenchymal stem cells [25]. Our findings showed that HSL was highly elevated in co-cultivated adipocytes compared to adipocytes cultured alone, coinciding with the result that adipocytes treated with co-cultivated medium exhibited a significant decrease in lipid accumulation.

Because adipose reversion is an important feature in the mechanism of metastasis, we want to continue studying what happens in adipocytes after their co-culture with breast cancer cells. In our study, the human adipokine chip test of the co-culture supernatant of MCF-7 cells and adipocytes revealed some altered proteins which are reported to be involved in the regulation of proliferation and metastasis of breast cancer cells, such as IGFBP-2, IL-6sR, IL-8, leptin, MIF, and TGF-β [26–31]. Among them IGFBP-2 and PDGF-BB both had an increase greater than 10-folds at the protein level in the supernatant. Based on the supernatant adipokine chip results, we chose 12 components that were up-regulated in MCF-7 CAA-CM for real-time RT-PCR analysis of adipocytes. IGFBP-2 and PDGF-BB had a 5.5-fold increase and an 8.8-fold increase at the mRNA level, respectively. These results support the conjecture that adipocytes can secrete some factors to enhance the migration ability of breast cancer cells. We intended to identify the most important soluble factor which was secreted from adipocytes and promoted the invasion ability of cancer cells after the reciprocal communication between the adipocytes and cancer cells. After extensively describing the changes in co-culture medium we chose IGFBP-2, PDGF-BB, IL-6sR and TNF-a for further assessments of their influence on metastasis. We found that IGFBP-2 enhanced the invasion ability of MCF-7 cells in vitro more prominently than did the other factors. It was coincidental that IGFBP-2 was a prominently increased factor in the co-culture medium of MCF-7 cells and adipocytes. According to previous studies [26,32], IGFBP-2 facilitates the migration of breast cancer cells in response to IGF signaling, and exogenous IGFBP-2 reduces phosphatase and tensin homolog deleted from chromosome 10 (PTEN) levels to protect MCF-7 cells against death. Chengcheng Guo et al. reported that plasma IGFBP-2 levels were significantly higher in stage IV than stage I or III lung cancer patients [26]. All of this evidence supports the concept that IGFBP-2 secreted from human adipocytes may promote breast cancer cell metastasis. Because our study found that MMP-2 is up-regulated at the mRNA level in MCF-7 cells treated with CAA-CM and MMP-2 is a proteinase which has been verified to promote cancer metastasis, we detected MMP-2 in MCF-7 cells treated with CAA-CM and CM plus with IGFBP-2. We found that MMP-2 was up-regulated at the protein level in both situations. The extent of the influence produced by CAA-CM was weaker than that of IGFBP-2 stimulation, possibly because it was an integrated effect influenced by many factors in CAA-CM. By combining the references and our results, we may infer that IGFBP-2 up-regulates MMP-2 and promotes metastasis.

To further confirm that the IGFBP-2 in CAA-CM was secreted by adipocytes, we knocked out IGFBP-2 signaling separately in the two cell types to do invasion assay and found that the IGFBP-2 derived from adipocytes exerts the major role. This phenomenon demonstrates that there was communication between adipocytes and breast cancer cells involving distinct signaling cascades in each partner and different molecular outcomes in each compartment. It is currently not very clear how the adipocytes can selectively increase the secretion of IGFBP-2, and we only confirmed that the IGFBP-2 secreted from adipocytes was very important to the invasion ability of breast cancer cells. The influence of IGFBP-2 was stronger than that of CAA-CM probably because these media also contained other tumor suppressing factors which are able to inhibit the IGFBP-2-induced invasion of MCF-7 cells. It is, therefore, conceivable that the IGFBP-2 induced increase in cancer cell invasiveness can be counteracted to some extent. However, because IGFBP-2 could create a microenvironment that is more favorable to invasion and metastasis through the up-regulation of MMP-2, the adipocytes may become a new target to reduce the extent of breast cancer metastasis. It is also likely that IGFBP-2 produced by these mature adipocytes contributes to the increased EMT in MCF-7 cells that is represented by the down-regulation of E-cadherin in our study.

The goals of our study were to investigate the effect of adipocytes on breast cancer cell motility and to determine if adipocytes can secrete some factors to promote this process. Next, we chose 10 metastatic and 10 non-metastatic pathologic samples to be stained by hematoxylin and eosin. Adipocytes around tumors that had broken through the basement membrane appeared smaller and more spindle-shaped, which indicates that these adipocytes has possibly become converted to fibroblast cells to some extent. This result was consistent with our in vitro result in which we found that PPAR-γ was decreased in adipocytes stimulated by CAA-CM. There was an increased amount of IGFBP-2 staining in both the tumor and surrounding adipose in the metastatic example (Fig. 4B). In another study, we have found that enough exogenous IGFBP-2 could stimulate endogenous IGFBP-2 expression in four breast cancer cells and different cell lines had different basal IGFBP-2 expression (data was not shown). So this may be a mechanism of self amplification in cancer cells communicating with microenvironment. In addition, MMP-2 was increased at the invading edge next with the fibroblast-like adipocytes. Thus, the communication between adipocytes and tumor cells increased IGFBP-2 secretion, activated MMP-2 in tumor cells and promoted the durable expression of MMP-2. These in situ results support the point of view that the indirect contact of cancer cells and adipocytes may change the function of adipocytes, which may in turn promote cancer cell invasion. Taken together, our results imply that breast cancer cells, once they traverse through the surrounding stroma, can subvert normal adipocytes to increase levels of IGFBP-2 in the microenvironment that then induce cancer cell migration, invasion, and an expanded metastatic pattern, thereby boosting the malignancy of breast cancer cells.

## Conclusions

In conclusion, our results demonstrate that an interaction between breast cancer cells and mature adipocytes induces an increase in the production of a number of molecules that are secreted from adipocytes and are capable of stimulating aggressive invasive behavior by breast cancer cells, even after the original interaction has ceased. Therefore, adipocytes are viable targets for the prevention of breast cancer metastasis and may become an adjuvant treatment for breast cancer. IGFBP-2 secreted by mature adipocytes plays a key role in this process, indicating that genetic alterations in the stroma surrounding the breast cancer may be a more accurate predictor of prognosis than whole tissue signatures, and further studies on targeting the secretion of IGFBP-2 from adipocytes will be more helpful in the development of anti-cancer therapeutics.
